# Nootropic Herbs, Shrubs, and Trees as Potential Cognitive Enhancers

**DOI:** 10.3390/plants12061364

**Published:** 2023-03-18

**Authors:** Matěj Malík, Pavel Tlustoš

**Affiliations:** Department of Agroenvironmental Chemistry and Plant Nutrition, Faculty of Agrobiology, Food and Natural Resources, Czech University of Life Sciences Prague, Kamýcká 129, 165 00 Prague, Czech Republic; malikmatej@af.czu.cz

**Keywords:** medicinal herbs, *Ginkgo biloba*, smart drugs, brahmi, learning ability, memory, gotu kola, Ayurvedic medicinal plants, antioxidant activity, *Panax ginseng*

## Abstract

Plant-based nootropics are a diverse group of natural drugs that can improve cognitive abilities through various physiological mechanisms, especially in cases where these functions are weakened or impaired. In many cases, the nootropics enhance erythrocyte plasticity and inhibit aggregation, which improves the blood’s rheological properties and increases its flow to the brain. Many of these formulations possess antioxidant activity that protects brain tissue from neurotoxicity and improves the brain’s oxygen supply. They can induce the synthesis of neuronal proteins, nucleic acids, and phospholipids for constructing and repairing neurohormonal membranes. These natural compounds can potentially be present in a great variety of herbs, shrubs, and even some trees and vines. The plant species reviewed here were selected based on the availability of verifiable experimental data and clinical trials investigating potential nootropic effects. Original research articles, relevant animal studies, meta-analyses, systematic reviews, and clinical trials were included in this review. Selected representatives of this heterogeneous group included *Bacopa monnieri* (L.) Wettst., *Centella asiatica* (L.) Urban, *Eleutherococcus senticosus* (Rupr. & Maxim.) Maxim., *Ginkgo biloba* L., *Lepidium meyenii* Walp., *Panax ginseng* C.A. Meyer, *Paullinia cupana* Kunth, *Rhodiola rosea* L., *Schisandra chinensis* (Turcz.) Baill., and *Withania somnifera* (L.) Dunal. The species are depicted and described, together with their active components and nootropic effects, and evidence of their efficacy is presented. The study provides brief descriptions of the representative species, their occurrence, history, and the chemical composition of the principle medicinal compounds, with uses, indications, experimental treatments, dosages, possible side effects, and contraindications. Most plant nootropics must be taken at optimal doses for extended periods before measurable improvement occurs, but they are generally very well tolerated. Their psychoactive properties are not produced by a single molecule but by a synergistic combination of several compounds. The available data suggest that including extracts from these plants in medicinal products to treat cognitive disorders can have substantial potential therapeutic benefits.

## 1. Introduction

Plant-based medicines and drugs have been used since ancient times to prevent and treat disease, but also to promote human mental well-being [1,2]. Those plants thought to strengthen and improve cognition belong to natural nootropics, and they formed a substantial part of the pharmacopeia available to ancient cultures and civilizations [3,4]. The term ‘nootropic’ was first used by Prof. Dr. Corneliu E. Giurgea in 1972/1973 to describe substances that primarily activate cognitive functions such as memory and learning, especially in situations where these functions are impaired [5,6]. The name comprises two Greek words, nöos, meaning ‘thinking,’ and tropein, which means ‘to guide’ [5,6,7]. Nootropic drugs affect cognitive function through various physiological pathways, but effects usually occur only after an extended time [8]. Pharmaceutical companies have invested large sums of money in synthesizing new compounds to alleviate or treat mental disorders. Still, formulas based on one single drug sometimes meet with limited success because of the multifactorial nature of such conditions.

To overcome this limitation and also to reduce the incidence of adverse side effects compared to conventional drugs, researchers have been attempting to meet the ever-increasing demand for nootropics by testing plant-based therapeutic products that can target multiple pathways either independently or in combination with conventional medications [9]. In this regard, using plant products based on traditional knowledge is currently on the rise in both developed and developing countries. Although there is considerable debate as to the effectiveness of these plant formulations based on sometimes questionable and unconvincing results, several controlled studies have demonstrated the positive effects of these natural substances on improving brain function [4,10,11].

This literature review provides an overview of the studied group of nootropic extracts from herbs, shrubs, and trees: a brief history, traditional uses, description, occurrence, chemical composition, validated assays, dosage, and side effects. This selection of plants was chosen for review and evaluation because a sufficient number of experiments, tests, and, where possible, clinical trials on their potential nootropic activities and positive effects on cognitive functions have been carried out. Original research articles, clinical trials, meta-analyses, systematic reviews, and relevant animal studies were included in this study. The review was not limited to experimental results but aimed to provide an up-to-date overview of commonly used over-the-counter supplements based on natural plant substances. Illegal plant drugs and those with a primarily non-nootropic function, such as vitamins, were not included.

## 2. Cognitive Impairment and Dementia

Before discussing plant effects on dementia and cognitive impairment, it is necessary to put them in the context of aging. There is still considerable confusion about what degree of memory loss is attributable to aging. Memory function, measured by the ability to acquire and store new information, is not significantly reduced in most older people. Studies have shown that when individuals who later developed dementia were excluded from an experimental group of otherwise healthy older people, there were only a few age-related declines and imbalances in cognitive functions, such as delayed recall. However, with the development of actual loss of memory due to the onset of neurological symptoms, there is often a loss of the capacity for self-reflection and self-assessment. Some individuals with incipient memory loss can tell that there has been a decline in their mental abilities. Still, most individuals with developing dementia are unaware that they are experiencing these dysfunctions [12,13].

The term dementia is sometimes replaced by the more descriptive term, serious neurocognitive disorder, which is not a single pathology but includes several clinical syndromes most often caused by some progressive neurodegenerative disease. The diagnosis requires proof of a cognitive decline that is not caused by another mental disorder and is severe enough to interfere with independent functioning in daily life. The most common type of dementia is Alzheimer’s disease (AD), a neurodegenerative disease characterized by the loss of neurons in specific brain areas. The most common symptoms are memory loss, reduced ability to acquire new information, and impairment of practical skills [14]. Other common types of dementia include vascular dementia, which is caused by problems with the blood supply to the brain that typically manifests as a series of minor strokes, and Parkinson’s disease, which is related to the loss of nerve cells in the part of the brain called the substantia nigra, that normally produce dopamine. The lack of dopamine causes the patient to gradually lose control of movement [15]. About half of the dementia cases are of a mixed type with multiple causes. Some dementia syndromes, such as vitamin deficiency or thyroid problems are reversible, but none of the common neurodegenerative dementias can be cured. Dementia is frequently preceded by a period of milder cognitive dysfunction [16].

## 3. Specific Nootropic Herb, Shrub, and Tree Species

The consumption of nootropic plant formulations is widespread and increasing, and the supply can no longer be covered only by collecting wild plants. Harvesting the relevant plant parts at the optimal time is essential because the content and spectrum of active components change during plant development [17]. In temperate zones, tree bark is harvested at the beginning of vegetative growth in early spring, while in the tropics, it can be harvested throughout the year. For collecting rhizomes and roots, the appropriate period for harvesting is during developmental dormancy, usually in spring or sometimes autumn. The stems and leaves are generally harvested just before or during blooming, whereas flowers are collected before they are fully developed, some even in the bud stage. Seeds and fruits are always harvested fully ripe [18,19]. The plant parts harvested at the most suitable time are air-dried and then subjected to further processing by various methods [20]. 

Medicinal preparations made directly from plant parts can be dried as a kind of ‘tea’ for water infusion or other types of extraction, added to food in powder form, or compounded into tablets or capsules. Active substances can be obtained from plant matrices by extraction, distillation, or pressing [21], and potentially beneficial compounds from plants, known as phytochemicals, continue to be an active area of research worldwide. Several species of herbs, shrubs, and trees have been selected for testing as nootropic agents because of their long history of use in traditional medicine. Research has already identified several promising natural substances that could act as cognitive enhancers, sometimes called ‘smart drugs’ [11,22,23].

### 3.1. Ashwagandha (Withania somnifera (L.) Dunal)

#### 3.1.1. History

*W. somnifera* is a highly prized herb in Ayurvedic medicine, where it has been used for more than 3000 years. It is also known as winter cherry, Indian ginseng, or by its Sanskrit name ashwagandha, which means ‘horsepower’ and is supposedly attributed to its pungent odor. Ashwagandha has been used for millennia as a ‘Rasayana’ (rejuvenator) for longevity. It was imported to Europe in the 16th century [24,25,26].

#### 3.1.2. Plant Description

**Figure 1:**
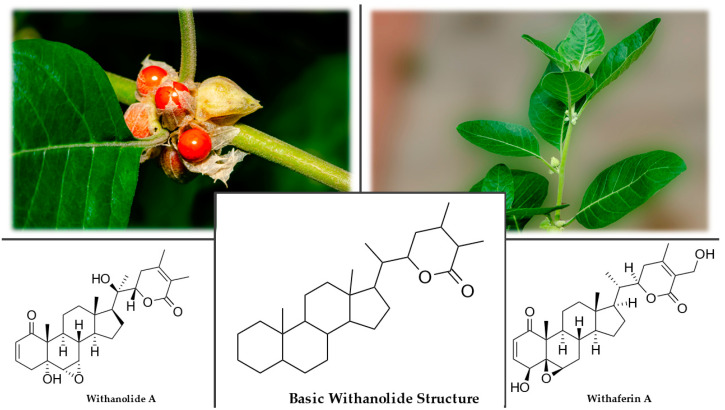
*Withania somnifera’s* main bioactive compounds, withanolides, and their chemical structures.

Ashwagandha is a perennial herb to low shrub from the Solanaceae family. The branched stem usually grows from 50 to 100 cm but can be 2.5 m tall. The hairy, bell-shaped, yellow-green flowers arise from the leaves’ troughs or at the shoots’ ends. The fruits appear as red berries wrapped in a calyx (Figure 1). The roots are smooth, 1–2 cm in diameter, and 30–40 cm long [24,27].

#### 3.1.3. Occurrence

Ashwagandha originated in North Africa but is widespread in Iraq, Pakistan, northern India, and China. *W. somnifera* is now also intensively cultivated by farmers in southern India. It is found in dry, almost arid, rocky places, along roads, on bushy savannas, and semi-deserts. Despite its significant medicinal potential, ashwagandha production is limited because there are not enough high-yielding species suitable for different agro-climatic conditions [27,28,29,30,31].

#### 3.1.4. Chemical Composition

The plant contains biologically active compounds such as alkaloids (isopelletierine, anaferine, anahygrine, withasomnine, cuscohygrine, ashwagandhine, and ashwagandhinine), sitoindosides, and acylsterylglucosides. It also contains steroidal compounds called withanolides, the triterpenoid lactones with an ergostane-based skeleton resembling ginseng’s active compounds (Figure 1). The first withanolide isolated was withaferin A, and the primary source of withanolides is the root. Because ashwagandha grows widely in diverse geographical areas, there are several different morphological forms differing in the content of pharmacological compounds [32,33].

#### 3.1.5. Uses and Specific Nootropic and Cognitive Effects of Ashwagandha

The roots of *W. somnifera* are the parts most frequently used, but occasionally the leaves are taken, dried, and infused into a tea. The fruit is sometimes used as an emetic [34]. Traditionally, ashwagandha is widely used as an adaptogen and recommended as a natural remedy for insomnia and neurological disorders [33,35].

Standardized extracts of *W. somnifera* have been shown to have multidimensional neuromodulatory effects in vitro and in animal models. The spectrum of proven results includes mitigating oxidative damage by strengthening the antioxidant defense system and increasing the expression of marker proteins responsible for nerve cells’ differentiation, growth, and communication. These specific effects of ashwagandha may be attributed to its potential for modulating neurotrophic factors, cytoskeletal elements, cell adhesion molecules, and synaptic proteins. Experimental evidence suggests that ashwagandha significantly modulates gamma-aminobutyric acid (GABA)-ergic, cholinergic, and antioxidant systems [36]. Based on mouse experiments, it was hypothesized that the neuroprotection of *W. somnifera* was mediated by a specific interaction with the GABA-gated chloride ionophore [37]. Another study on rats also provided direct evidence of the GABAergic activity of this herb on mammalian ionotropic GABA receptors [38].

Withanolides showed inhibitory potential in vitro against the activity of the acetylcholinesterase enzyme and induced amyloid-β aggregation. In addition, they demonstrated dose-dependent spasmolytic and Ca^2+^ antagonist activities in isolated preparations of rabbit jejunum. This may be useful in preventing excessive elevation of intracellular neuronal calcium and prolonging cell survival and function [39]. Chronic administration of *W. somnifera* root extract (100 and 200 mg/kg) to mice, on the contrary, restored levels of acetylcholinesterase activity in the cortex, hippocampus, and striatum. Experiments showed that ashwagandha increased the numbers of platelets, red and white blood cells, as well as the hemoglobin content. The increase in the number of red blood cells resulted in an increase in the blood’s ability to transport oxygen to the peripheral system, which ensured a greater maximal aerobic capacity. This combination of effects makes them promising candidates for further studies to treat Alzheimer’s disease and related problems [40].

Administration of ashwagandha root extracts to rats for two weeks dose-dependently improved behavioral, biochemical, and enzymatic changes induced by 3-nitropropionic acid. Intraperitoneal administration of 3-nitropropionic acid caused body weight loss, decreased motor functions, depleted antioxidant enzyme levels, blocked adenosine triphosphate synthesis by inhibiting mitochondrial complex activity in various brain regions, and significantly increased lipid peroxidation and lactate dehydrogenase enzyme levels [41]. In another study, a rat model of tardive dyskinesia was developed by injections of reserpine. Lipid peroxidation products and oxidative stress are involved in the pathophysiology of this disease, and long-term administration of ashwagandha root extract reduced glutathione levels and lipid peroxidation and reversed the decline in brain superoxide dismutase (SOD) and catalase levels. These findings suggest that the neuroprotective effects are partially mediated by antioxidant activity [42]. In addition, it also alleviated memory impairment by prolonging acquisition latency as well as retention of transfer in propoxur-treated rats [43]. Thus, studies in rats showed ashwagandha’s potential in treating Huntington’s disease.

In a double-blind, placebo-controlled clinical trial, *W. somnifera* root extract was evaluated for its cognitive potential in euthymic bipolar subjects. The extract demonstrated significant efficacy in improving the patient’s performance of cognitive tasks [44]. In another clinical trial, ashwagandha extract administered daily for 30 days at doses of 225 or 400 mg improved cognitive flexibility, visual memory, psychomotor speed, reaction time, executive function, and stress response [45]. Acute supplementation of 400 mg of a proprietary *W. somnifera* root and leaf extract also helped maintain attention and increased short-term or working memory in healthy young adults [46]. In a double-blind, placebo-controlled trial, patients with anxiety disorders were given an ethanol extract of ashwagandha daily for 2–6 weeks. The extract was well tolerated, did not cause any side effects, and showed a significant anxiolytic effect [47]. The daily dose should be 6 to 10 g of ground root or the equivalent of 750 mg to 1250 mg of *W. somnifera* extract [48].

*W. somnifera* is an important source of many potentially pharmacologically and medicinally valuable phytochemicals such as withanolides, sitoindosides, and various beneficial alkaloids. Although the observed nootropic effects of ashwagandha are promising for using the herb to improve cognitive dysfunction, there are limitations due to gaps in the current literature. While *W. somnifera* has been successfully used in Ayurvedic medicine for centuries, there is still a lack of accurate clinical data to support its therapeutic use. Ashwagandha extracts are effective in isolation but may also have synergistic effects when administered in combination with other herbal or synthetic drugs. One should also not assume that the pro-cognitive effects are generalizable across all disease states because the different etiologies can involve different mechanisms. However, the available data seem promising. It would be worthwhile to conduct a methodologically precise and rigorous future clinical trial with a large and well-defined sample to obtain more meaningful empirical results.

#### 3.1.6. Side Effects and Contraindications

Ashwagandha is generally considered safe, and the acute median lethal dose (LD_50_) of total alkaloids from roots was 465 mg/kg body weight in rats and 432 mg/kg body weight in mice [49]. A subacute study of ashwagandha extract on rats showed no significant disturbances of biochemical, hematological, or histopathological parameters in vital organs [50]. *W. somnifera* is best taken in the evening because, as already mentioned above, it is traditionally used for sleep disorders and can therefore act as a sedative in large doses. Possible overdose can also cause gastrointestinal problems and vomiting, so treatment should be started with small doses and gradually increased. Ashwagandha is not recommended during pregnancy or in cases of hyperthyroidism [51]. Chronic stress increases cortisol levels leading to lower levels of triiodothyronine and thyroxine. *W. somnifera* is believed to stimulate the endocrine system and increase thyroid hormone levels by reducing cortisol. However, this also means that ashwagandha can worsen the symptoms of hyperthyroidism, potentially leading to a serious form of hyperthyroidism called thyrotoxicosis [52,53].

### 3.2. Asiatic Pennywort (Centella asiatica (L.) Urban)

#### 3.2.1. History

*C. asiatica* is also known in China as gotu kola and is an important medicinal herb used in traditional Asian medicine for thousands of years [54,55].

#### 3.2.2. Plant Description

**Figure 2:**
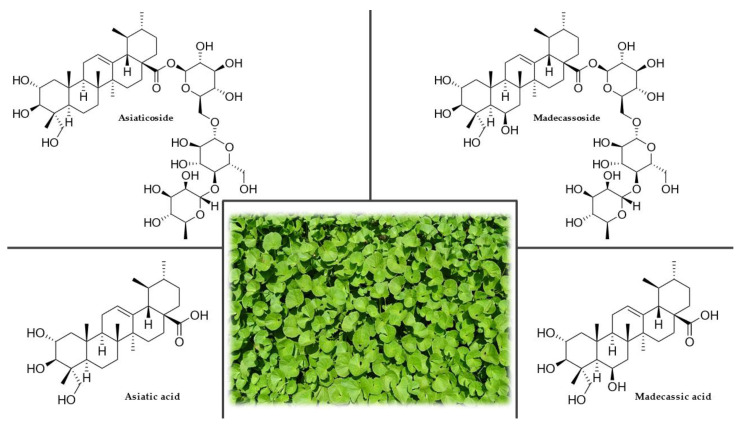
*Centella asiatica’s* main bioactive compounds and their chemical structures.

*C. asiatica* is an evergreen, creeping, ground-cover plant belonging to the Apiaceae family with stems up to 1.5 m long that reproduces by producing stolons (Figure 2). Its roots are covered with nodules. It has kidney-shaped, shallowly serrated leaves up to 30 cm long. Tiny, white or red flowers appear in the axils of the leaves, arranged in groups of two to five into an umbel. The fruit is spherical and has raised ribs [54,56,57,58].

#### 3.2.3. Occurrence

*C. asiatica* grows on the edges of streams, swamps, and wet pastures, in tropical and subtropical areas around the world, up to rocky regions at an altitude of 1900 m above sea level. It occurs mainly in Southeast Asia, India, Sri Lanka, China, the western Pacific Islands, Madagascar, South Africa, the southeastern United States, Mexico, Venezuela, Colombia, and the eastern part of South America [59,60,61,62].

#### 3.2.4. Chemical Composition

The highest concentration of phytochemicals is found in the leaves, with lesser amounts in the petioles and roots. The main active compounds (Figure 2), which occur freely in the plant, are triterpene acids (asiatic, madecassic, thankunic, and isothankunic acids) or their heteroglycosides (asiaticoside, madecassoside, braminoside, brahmoside, brahminoside, thankuniside, and isothankuniside). The quantitative and qualitative levels of individual compounds depend on the plant’s location and time of collection. Other constituents present in the plant are flavonoids (quercetin, kaempferol, astragalin, rutin, apigenin, and naringin), phytosterols (stigmasterol and β-sitosterol), essential oils, polyacetylenes, coumarins, amino acids, heterocyclic compounds, tannins, and resins. *C. asiatica* is also rich in vitamin C, vitamin B_1_, vitamin B_2_, niacin, and β-carotene. The ash contains chloride, sulfate, phosphate, calcium, magnesium, sodium, potassium, and iron [63,64,65].

#### 3.2.5. Uses and Nootropic or Cognitive effects of Gotu Kola

The entire plant, including the leaves, stems, and roots, is traditionally consumed for its therapeutic value. In Asian countries where this plant originates, the fresh leaves are eaten raw or cooked as a vegetable. It is also part of drinks or curry spice mixtures [66]. The herb is also commonly used in Sri Lanka as part of a nutritional porridge for feeding pre-school children known as *kola kenda*, which is a mixture of gotu kola, rice, and vegetables [67].

An ethanol extract of gotu kola mediated protection against amyloid-β-induced aggregate neurotoxicity by modulating the antioxidant defense system in cells in vitro, including the activity of catalase, SOD, glutathione peroxidase, glutathione reductase, and levels of glutathione and glutathione disulfide. Amyloid-β is one of the main components of neurofibrillary tangles and senile plaques found in the brains of patients with AD [68]. To verify the stimulating effects of gotu kola on the central nervous system (CNS), treatment with an aqueous extract was compared to the effects of chloroform and methanol extracts on the behavior of mice. Only the aqueous extract of the whole plant at a dose of 200 mg/kg for 14 days showed improvement in learning and memory. At doses of 200 and 300 mg/kg of an aqueous extract of *C. asiatica*, a significant increase in glutathione levels and a decrease in malondialdehyde concentration in the brain were found. Furthermore, at 300 mg/kg, an increase in catalase activity was observed, but the SOD level was not significantly changed [69]. However, daily oral administration of *C. asiatica* extract (50 mg/kg) to mice for 14 days increased the concentration of antioxidant enzymes (glutathione peroxidase, catalase, and SOD), glutathione, and ascorbic acid [70]. Evaluation of the antioxidant activity of the extracts of this herb and their comparison with natural (α-tocopherol) and synthetic (BHT, butylated hydroxytoluene) antioxidants showed that phenolic substances are the main contributors to the antioxidant activity. From 3.23 to 11.7 g of phenolic substances per 100 g of dry sample were found in the extract [71].

Another experiment showed a reduction in lipid peroxidation dependent on the supplementation of gotu kola extract in aged mice. A 300 mg/kg extract dose for 60 days resulted in a significant reduction in lipid peroxidation in the cerebral cortex, thus, demonstrating a strong potential neuroprotective effect of the extract against damage to brain tissue [72]. An in vivo study in mice revealed that increased nitric oxide (NO) production might be involved in the mechanism of the protective effect of gotu kola against anxiety caused by sleep deprivation, neuroinflammation, or oxidative damage [73]. The gotu kola plant contains multiple active fractions that increase neurite extension in vitro and accelerate axonal regeneration in male rats after oral administration. This suggests that components in *C. asiatica* may help accelerate the repair of damaged neurons [74].

A subsequent study evaluated the nootropic activity in 3-month-old mice given 200, 500, 700, and 1000 mg/kg aqueous extract of *C. asiatica* for 15 days. Learning ability, maze orientation, and acetylcholinesterase activity were assessed. The treatment resulted in increased dendritic arborization of hippocampal CA3 neurons and hippocampal acetylcholinesterase activity. Administration of the extract also affected the morphology of neurons and improved learning ability and brain function [75]. In vivo experiments on rats in maze and cage tests, monitoring their locomotor activity and social interactions, have also shown that gotu kola extracts and pure asiaticoside had anxiolytic activity. Asiaticoside did not significantly affect locomotor activity, suggesting that this compound does not have sedative effects [76]. 

A clinical study of 33 volunteers evaluated the effects of a 70% ethanol extract of *C. asiatica* on general anxiety disorder. The results showed that *C. asiatica* significantly alleviated anxiety-related disorders and reduced stress and depression [77]. A randomized, placebo-controlled, double-blind trial examined the effect of this herb on cognitive function in healthy elderly volunteers. A high dose of *C. asiatica* extract was shown to improve working memory, suggesting its potential for mitigating age-related cognitive decline [78]. The daily dose of gotu kola should be approximately 600 mg of dried leaves or 60–120 mg of a standardized extract containing at least 85% triterpenoid glycosides [79,80].

*C. asiatica* exhibits remarkable nootropic, cognitive, and neuroprotective properties. These actions support and emphasize its potential to modulate disease processes and conditions associated with neurodegenerative disorders; however, more extensive knowledge of the active components of this herb is still needed. The triterpenoid glycosides such as asiaticoside and madecassoside, and triterpenoid acids such as asiatic and madecassic acid have been shown to contribute to the neurological effects, and *C. asiatica* extracts tend to be standardized for the content of these compounds. However, further detailed chemical analyses using modern methods and approaches could identify other bioactive compounds. It is also necessary to further investigate the interactions between different compounds because the multiple components in broad-spectrum extracts can simultaneously influence several pathways. Adequate chemical characterization and standardization in these experiments are essential to enable comparison between studies. Currently, there is insufficient clinical evidence to confirm the effect of *C. asiatica* on improving cognitive functions. Therefore, well-designed future clinical trials are needed to assess its safety and the impact of a standardized dose of *C. asiatica* or its extract on cognitive function and mood.

#### 3.2.6. Side Effects and Contraindications

When regular doses are administered orally, *C. asiatica* is very well tolerated. The safety of consuming the dried plant has been demonstrated by toxicity testing in which the LD_50_ of dried powder administered orally to mice was more than 8 g/kg. No interactions with other drugs are known. In rare cases, patients may experience nausea, vomiting, itching, and photosensitivity. Locally, it can cause allergic contact dermatitis. Although no teratogenic effects have been reported, it should not be used during pregnancy or breastfeeding [81,82,83].

### 3.3. Eleuthero (Eleutherococcus senticosus (Rupr. & Maxim.) Maxim.)

#### 3.3.1. History

In connection with the search for substitutes for ginseng, which is difficult to grow, some other plant species from the Araliaceae family were studied in the 1950s. *E. senticosus*, also popularly called devil’s bush or Siberian ginseng, has occupied an important position in China, where it has been used for over 5000 years [84,85].

#### 3.3.2. Plant Description

**Figure 3:**
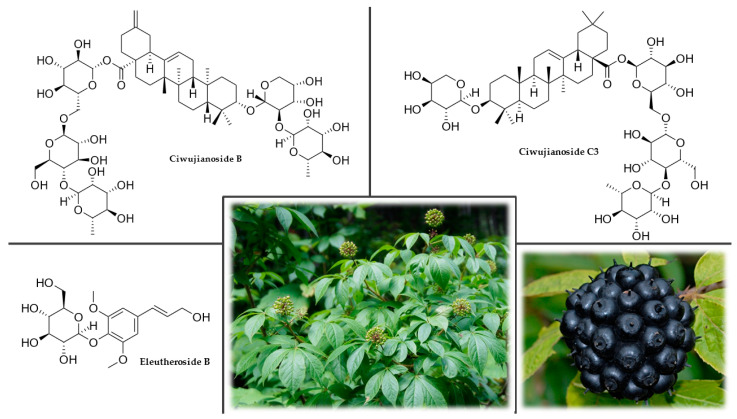
*Eleutherococcus senticosus’s* main bioactive compounds, ciwujianosides and eleutherosides, and their chemical structures.

Eleuthero is a shrub growing to a height of 2–5 m with dense, thorny branches and bark on young shoots of a yellow-brown color. The leaves are long, petiolate, five-fold, 6–12 cm long, and 3–7 cm wide. They have an elongated or elliptical shape with a sharply pointed apex, serrated at the edges, hairy at the veins, or with sparse spines. The small flowers form spherical inflorescences with pink and cream petals. The fruit is a black spherical berry containing 2–7 seeds (Figure 3). Eleuthero has a highly developed root system that is usually horizontally distributed in the upper layer of the soil. A relatively low reproductive capacity also characterizes it. Both generative and simple vegetative reproduction are ineffective. The most efficient method to increase its reproductive potential for cultivation is in vitro techniques [84,86,87,88].

#### 3.3.3. Occurrence

It occurs naturally in the taiga of the Far East. It is widespread in Primorsky Krai, Khabarovsk Krai, Amur, and southern Sakhalin. In addition to these areas, it also grows in Korea, Japan, and northeastern China. It primarily forms a continuous understory in mixed and coniferous forests but also occurs in clearings up to an altitude of 800 m. Sunny habitats and soils sufficiently supplied with water are ideal [84,86].

#### 3.3.4. Chemical Composition

The main constituents are eleutherosides and ciwujianosides (Figure 3), which in most cases, belong to the group of heteroglycosides. Other important components are polysaccharide glycans (eleutherans A, B, C, D, E, F, and G), hydroxycoumarins (isofraxidine), lignans (sesamin), flavones, phytosterols (daucosterol and β-sitosterol) and essential oils (α-bergamotene, δ-elemene, β-elemene, γ-cadinene, α-pinene, and (+)-aromadendrene). Dried fruits, consumed as food, are rich in calcium, magnesium, manganese, zinc, and copper [86,89,90].

#### 3.3.5. Uses and Nootropic or Cognitive Effects of *Eleutherococcus senticosus* (Rupr. & Maxim.) Maxim.

The root is ground into a powder and taken in capsules or as a tincture. A decoction known as Siberian ginseng tea is made from dried leaves [84,91].

In an in vitro study, an essential oil produced from eleuthero seeds showed concentration-dependent reducing power and significant inhibition of ferric-ion-induced lipid peroxidation. It has also demonstrated substantial antioxidant activity by inhibiting NO, 1,1-diphenyl-2-picrylhydrazyl (DPPH), superoxide, and hydroxyl-free radicals [92]. According to the results of another in vitro study, *E. senticosus* could be used in antioxidant therapy as an agent capable of reducing the toxic effects of free radical products of oxygen reduction in the body [93]. 

An in vitro experiment investigated the effects of eleuthero extracts on neurite regeneration and synapse reconstruction in cultured rat cortical neurons damaged by amyloid-β_25-35_. Treatment with aqueous or methanol extracts significantly reconstructed neuronal synapses and promoted axonal and dendritic regeneration [94]. A similar in vitro study was performed with an aqueous extract of *E. senticosus* leaves. Using an axonal density assay, this extract successfully restored neurite growth after amyloid-β25-35-induced degeneration [95]. Thus, eleuthero extracts have been confirmed to protect against neuritic atrophy and cell death from amyloid-β_25-35_ treatment. One of the essential active compounds may be eleutheroside B [94,95].

One study in mice showed that oral administration of eleuthero leaf extract improved memory, while ex vivo, it was confirmed that the bioactive saponin compounds, eleutheroside M, ciwujianoside B, and ciwujianoside C3, could penetrate the blood–brain barrier to act on the brain. These three compounds separately and in the complex extract elicited dendritic elongation of primary cultured cortical neurons, which may be related to memory improvement [96]. Based on memory and learning tests in rats, an aqueous extract of the saponin fraction of dried eleuthero leaves had a significant reinforcing effect. In addition, molecular docking experiments demonstrated that key BBB-penetrating saponins primarily interacted with targets of mitogen-activated protein kinase 1 (MAPK1) and MAPK8 to produce a neuroprotective effect [91].

Twenty volunteers over 65 years of age with hypertension were subjected to a randomized, double-blind study with an eleuthero extract at a dose of 300 mg/day for eight weeks. The results indicated that *E. senticosus* improved some aspects of mental health and social functioning and was safe to use [97]. Another randomized, placebo-controlled, double-blind trial on healthy volunteers evaluated the cognitive enhancement and anti-stress effects of the combined extracts of *E. senticosus* leaf and *Drynaria fortunei* rhizome. The intake period was 12 weeks, and the Japanese version of the Repeatable Battery for the Assessment of Neuropsychological Status was used for neurocognitive assessment. The results revealed that the combined treatment with these two herbal extracts reduced anxiety and improved language and cognitive function in healthy adults with no side effects [98]. The recommended dose of eleuthero is 2–3 g of dried root or an equivalent amount of extract daily [99]. A standardized extract from the roots and rhizomes is prepared at a ratio of 1:12, so 10 mg of the extract is approximately equivalent to 120 mg of crude herb [100,101].

*E. senticosus* appears to have a more substantial biological effect during periods of increased stress. The data also suggest that ciwujianosides B and C3 and eleutheroside M are likely the main active compounds in eleuthero leaf extracts responsible for neuronal activation. Further research is needed to determine the most appropriate treatment regimen for cognitive enhancement. Despite these limitations, *E. senticosus* appears to be a safe and well-tolerated herbal medicine, but further rigorous clinical trials are needed to confirm these findings.

#### 3.3.6. Side Effects and Contraindications

No serious side effects have been reported with the use of *E. senticosus*. Therefore, it can be considered a relatively safe herbal medicine [97,98]. It slightly increases blood pressure, so its use by patients with hypertension is not recommended [102].

### 3.4. Ginkgo (Ginkgo biloba L.)

#### 3.4.1. History

Ginkgo, which is related to the ferns and conifers, is one of the oldest plant species still existing on Earth. It flourished from 250 to 270 million years ago and is therefore sometimes referred to as a living fossil. The Ginkgoales order of plants excelled in species richness from the Triassic to the Cretaceous, but today it is represented by only a single species, *G. biloba*. In the 10th century, Chinese monks began planting ginkgo in monastery gardens and chewing its leaves to keep them mentally alert. This species is so hardy that one tree even survived the atomic bomb explosion in Hiroshima, so the Japanese consider ginkgo a symbol of hope. The plant came to Europe in 1727, where it continues to be grown without significant problems [103,104,105,106].

#### 3.4.2. Plant Description

**Figure 4:**
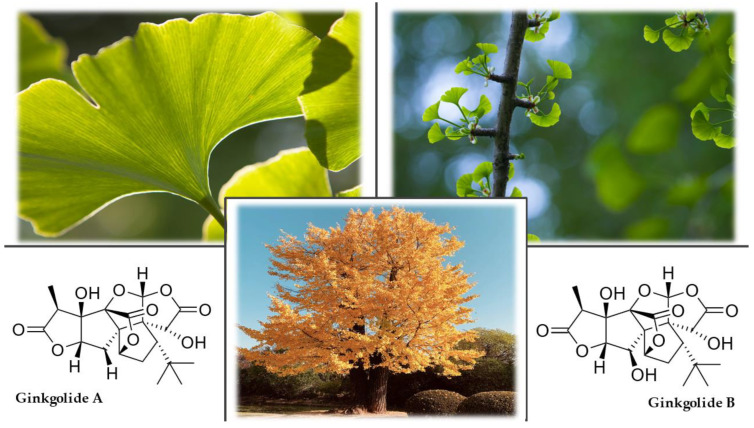
*Ginkgo biloba’s* main bioactive compounds, ginkgolides, and their chemical structures.

The ginkgo is a deciduous tree that grows to 40 m with heavily furrowed, cracked bark. It can live up to one thousand years. Its unmistakable fan-shaped leaves have long petioles and are notched or lobed. In autumn, the leaves turn a bright golden-yellow color (Figure 4). The flowers are dioecious and are relatively inconspicuous. In male plants, an inflorescence is formed by coniferous formations that look like catkins. In female plants, the inflorescence consists of small green panicles on stalks. The fruit is a stone fruit (drupe) with a diameter of 18–25 mm, initially green but turning yellow on the surface when ripe. The seed’s outer shell contains butyric acid that gives it a characteristic strong, disagreeable odor [106,107].

#### 3.4.3. Occurrence

Ginkgo comes from a small area in Chekiang province in southeastern China. It is a relatively undemanding plant and is grown all over the world. It tolerates an urban situation well and is often planted in parks and along streets. It prefers well-drained, permeable soils rich in nutrients and tolerates an acidic to alkaline pH [103,105].

#### 3.4.4. Chemical Composition

The active compounds are present mainly in seeds and leaves. Ginkgo seeds contain minerals, flavonoids, triterpene lactones, phenolic compounds, ginkgotoxin (4-*O*-methylpyridoxine), ginkgolic acid, and ginnol, which inhibit the growth of fungi and bacteria. These compounds have astringent effects. Flavone glycosides, biflavones, triterpene lactones (ginkgolides A, B, C, J, M, and bilobalide), alkylphenols, proanthocyanidins, polyprenols, phenolic acids, 6-hydroxykynurenic acid, and ginkgotoxin are found in the leaves [107,108,109].

#### 3.4.5. Uses and Nootropic and Cognitive Effects of *Ginkgo biloba* L.

The leaves are harvested from spring to autumn, dried and ground, and used to produce extracts and tinctures [110]. The ripe fruits should only be consumed roasted or boiled because their harmful cyanide and amygdalin content is destroyed by heating [111]. Hulled and roasted seeds can be safely eaten [109].

In an in vitro study, the ability of a standardized extract from the leaves of *G. biloba* (EGb 761) to quench free peroxyl radicals, which are widely involved in lipid peroxidation, was evaluated on liposomes and human lipoproteins. The extract demonstrated a protective effect against oxidative damage in all tested systems, suggesting that it is an effective peroxyl radical scavenger [112]. Middle-aged (12-month-old) female rats were given an intraperitoneal injection (10 mg/kg body weight) of aluminum and an oral dose (100 mg/kg body weight) of ginkgo extract daily for six weeks. Administration of the extract also significantly reduced reactive oxygen species (ROS), lipid peroxidation, NO, and citrulline levels and increased reduced glutathione and antioxidant enzyme activity compared to non-treated control rats [113].

The results of an ex vivo experiment on rats showed that ginkgo extract had specific neuroprotective effects that could be useful in treating chronic cerebral hypoperfusion. The pharmacological mechanism of the extract involved modulation of the cholinergic system and inflammatory mediators [114]. In mice, the effect of EGb 761 on synaptic transmission and plasticity in the hippocampus, which is a crucial area for memory development, was evaluated. A 30-day administration of the extract to mice improved neuronal plasticity in the hippocampus, probably by a direct effect on glutamate metabolism [115]. Incubating a line of mutated neuroblastoma cells with EGb 761 inhibited the formation of amyloid-β fibrils involved in AD development. The extract also attenuated mitochondrial-initiated apoptosis and reduced the activity of caspase-3, an enzyme involved in the apoptotic cascade [116].

In a randomized, placebo-controlled, multicenter study, 216 patients with multi-infarct, pre-senile, and senile degenerative dementia of the AD type were enrolled for a 24-week treatment period with EGb 761. Patients receiving a daily oral dose of 240 mg of EGb 761 showed significant improvement compared to the placebo group [117]. In another double-blind, placebo-controlled clinical trial, volunteers were given neuropsychological tests before and after treatment with *G. biloba* extract. They showed significant improvements in information processing speed and working memory [118]. Another study evaluated the efficacy and tolerability of a six-month administration of ginkgo to patients with vascular dementia, early AD, or mixed dementia, which included both dementia types. The results confirmed the benefits of *G. biloba* extract treatment, which was very well tolerated [119]. On the opposite side, however, a review critical of the data from several randomized clinical trials which did not provide convincing evidence that ginkgo preparations or extracts used for short or long periods positively affected cognitive performance in healthy people under the age of sixty [120]. A randomized, double-blind, placebo-controlled clinical trial of patients aged 70 years or older who spontaneously reported memory problems showed that long-term use of EGb 761 did not reduce the risk of progression to AD compared with placebo [121]. The recommended daily dose of EGb 761 is from 120 to 300 mg, standardized to a content of 24% flavone glycosides and 6% terpene lactones [122,123,124]. The standardized extract of *G. biloba* is already widely used as a dietary supplement or as a support agent in treating several adverse conditions, memory and concentration problems, depression, anxiety, confusion, and headaches. The supposed mechanisms of action reflect the properties of several compounds in the extract and primarily include antioxidant activity by reducing free oxygen radicals, increasing blood flow by dilating blood vessels, reducing blood viscosity, and modifying neurotransmitter systems. *G. biloba* also appears relatively safe with no serious adverse side effects. Despite all this, the evidence regarding predictable clinical benefits for patients with dementia or cognitive impairment is still inconsistent and unreliable.

#### 3.4.6. Side Effects and Contraindications

Among the mild side effects that may occasionally occur, especially with excessive consumption of *G. biloba*, are headaches, allergic skin reactions, palpitations, and gastrointestinal problems. Since it causes blood thinning, it should not be taken before a planned surgical procedure. Its use also requires caution in patients with bleeding disorders, on anticoagulant therapy, or those taking nonsteroidal anti-inflammatory drugs. In patients with seizure disorders, the ginkgotoxin found primarily in the seeds may lower the threshold for an epileptic seizure initiation associated with vitamin B_6_ deficiency. Ginkgotoxin is structurally related to vitamin B_6_, so it can compete with it and therefore interfere with its metabolism, function, and biosynthesis. Vitamin B_6_ is a coenzyme of glutamate decarboxylase, but ginkgotoxin competitively inhibits the production of the suppressive neurotransmitter GABA. Thus, in the case of vitamin B_6_ deficiency, ginkgotoxin may be responsible for epilepsy because CNS neurons become abnormally excitable due to reduced GABA production. So far, no data have confirmed the safety of ginkgo use in infants and pregnant and lactating women [125,126,127,128,129,130].

### 3.5. Ginseng (Panax ginseng C.A. Meyer)

#### 3.5.1. History

*Panax ginseng*, commonly called ginseng, which in Korean means ‘the root of life,’ is an ancient part of the East Asian materia medica. It was imported into Europe at the beginning of the 9th century. In the 18th century, it was sold in pharmacies as a medicine to preserve health and youth, and especially as an aphrodisiac. The price was calculated according to the dry or fresh weight and often amounted to three or more times its weight in silver or gold. Due to the similar shape of its root to the human body, it was ascribed a miraculous power and was sometimes worn as a talisman. The genus name, *Panax*, comes from the Greek words *pan*, which means ‘everything,’ and *akos,* meaning ‘medicine.’ Ginseng root extract still maintains its premier place among plant drugs in traditional medicine [131,132,133].

#### 3.5.2. Plant Description

**Figure 5:**
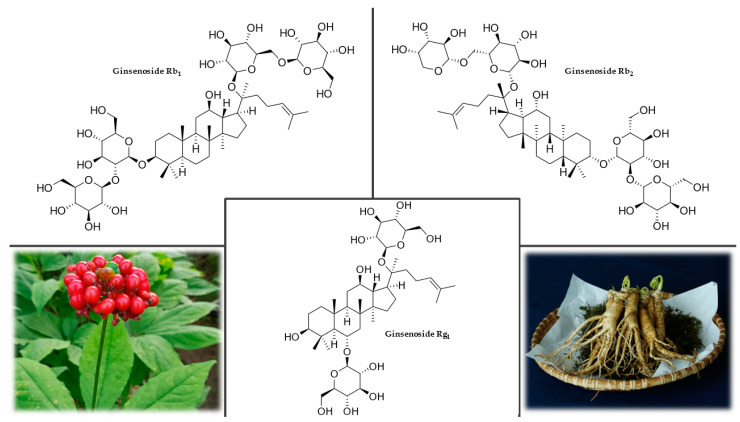
*Panax ginseng’s* main bioactive compounds, panaxosides (ginsenosides), and their chemical structures.

Ginseng is a perennial plant with a thick root, usually divided into upper and lower parts, somewhat resembling a human figure. A 30–60 cm high stem grows from the root, with 3–4 petiolate and palmate-pinnate leaves consisting of five marginally serrated leaflets. The tiny, usually inconspicuous flowers resemble ivy flowers and are arranged in an umbel. The fruits are spherical red berries (Figure 5). Depending on the natural conditions and the cultivation method, this species is usually divided into three forms: cultivated ginseng, ginseng grown in mountain conditions, and wild mountain ginseng. Cultivated ginseng makes up the majority of the product available on the market. Ginseng grown in forests and mountains tends to achieve the qualitative characteristics of wild mountain ginseng. Wild ginseng is rare and is always one of the most expensive commodities on the medicinal plant market [134,135].

#### 3.5.3. Occurrence

This plant originated in Korea but is currently widespread, mainly in mountainous areas from Nepal to Manchuria and from eastern Siberia to Korea. It grows readily in mountain forests on brown soils or podzols as long as it receives constant and relatively high humidity and uniform temperatures without sharp fluctuations between day and night [134,136].

#### 3.5.4. Chemical Composition

Ginseng root contains a wide range of biologically active substances, namely polysaccharides (panaxan A-U), essential oils, amino acids (tyrosine, leucine, serine, and arginine), peptides, flavonoids, minerals, vitamins (B_1_, B_2_, B_12_, and C), triterpenoids (dammarane), and saponins, referred to as ginsenosides (or more appropriately panaxosides). Panaxosides (Figure 5) are the major pharmacologically active compounds and account for a significant proportion of the weight of dried ginseng, typically more than 5%. According to the aglycone, it is divided into three main groups: protopanaxadiol-, protopanaxatriol-, and oleanane-type saponins [137,138]. A high content of potassium, phosphorus, calcium, sodium, iron, sulfur, and some microelements characterize the mineral composition of ginseng roots. The plant contains a significant amount of aluminum, silicon, manganese, magnesium, barium, titanium, and strontium. Ginseng root also contains fats and various unidentified substances [139,140].

#### 3.5.5. Uses and Nootropic or Cognitive Effects of *Panax ginseng* C.A. Meyer

Ginseng roots can be processed in different ways, affecting the content of active compounds and the degree and spectrum of their medicinal effects. When roots are peeled and dried, they are called white ginseng. Alternatively, the ginseng roots can be steamed without peeling, after which they are referred to as red ginseng [141]. To extend the shelf life, the steaming process is followed by drying. However, dried ginseng has disadvantages when used in traditional cuisines because of its tough fibrous texture after rehydration. Grinding to a powder or preparing an extract are alternatives to overcome this problem [142].

The results of an in vitro study showed that purified ginsenoside Rb_1_ induced NO production in cultured human aortic endothelial cells, which may be responsible for ginseng’s hypotensive and vasorelaxant effects [143]. Experiments on five-day-old male chickens suggested that ginsenoside Rb_1_ improved memory in a visual discrimination task and that the nootropic effect was also associated with changes in anxiety [144]. Another work examined the anti-neuroinflammatory effects of ginsenoside Rb_1_ in a rat model of AD. Ginsenoside Rb_1_ reversed the changes in several markers of neuroinflammation in the hippocampus. In the future, this compound could be used as a therapeutic agent for patients with memory impairment [145]. Another study determined the neuroprotective effects of six different ginsenosides. Ginsenoside Rb_2_ was found to improve the viability of HT-22 hippocampal mouse neurons against glutamate-induced neurotoxicity. Treatment with ginsenoside Rb_2_ reduced glutamate-mediated Ca^2+^ influx and intracellular ROS accumulation, subsequently suppressing MAPK activation caused by glutamate-mediated oxidative stress and apoptosis-inducing factor-mediated apoptotic cell death. The study demonstrated that ginsenoside Rb_2_ rescued cells damaged by ischemic brain injury and showed neuroprotective efficacy in an animal model [146]. Supplementation with ginsenoside Rg_1_ improved the performance of aged mice in a behavioral test by significantly increasing the expression of proteins associated with synaptic plasticity in the hippocampus, including synaptophysin, *N*-methyl-D-aspartate receptor subunit 1, the α-isoform of calcium-calmodulin-dependent protein kinase II and postsynaptic density protein 95, by promoting activation of the mammalian target of rapamycin (mTOR) pathway [147]. The impact of Korean red ginseng extract on the dissociation and aggregation of tau protein in vitro was also evaluated. Analysis revealed that the extract inhibited aggregation and promoted the dissociation of tau aggregates [148]. Tau is a microtubule-associated protein normally expressed in mature neurons. However, abnormally hyperphosphorylated tau dissociates from microtubules and self-aggregates. These tau aggregates promote neuronal dysfunction and can ultimately lead to the death of neurons. Therefore, suppressing the aggregation or stimulating the dissociation of tau aggregates has been proposed as an effective strategy for treating neurodegenerative diseases such as frontotemporal dementia or AD [149]. 

In a seven-day experiment on rats, oral administration of extracts from *P. ginseng*, *G. biloba,* and a combination of the two caused changes in behavior consistent with significant memory improvement. Individual doses of antagonists and agonists of different subtypes of serotonin receptors influenced the behavioral effects of the tested drugs and the ability to maintain the learned behavior. The results indicated the involvement of the serotonergic transmitter as an important neurochemical correlate of the behavioral and memory effects of the study drugs. This involvement was likely modulated by differences in the functionality of the serotonin receptor subtypes [150]. 

A double-blind, randomized trial investigated the antioxidant effects of ginseng treatment over four weeks in 82 volunteers. Ginseng supplementation led to a decrease in serum levels of ROS and malondialdehyde. Glutathione content and glutathione reductase activity increased in the group receiving 2 g of ginseng daily. No significant changes in total antioxidant capacity or peroxidase, catalase, or SOD activities were observed. Ginseng enhanced antioxidant defenses in a healthy population and thus could be used as a potential antioxidant supplement [151]. According to the results of a randomized, double-blind, placebo-controlled clinical trial involving 90 volunteers, it was found that daily oral administration of 3 g of ginseng powder for six months positively improved cognitive functions, especially visual memory, in subjects with mild cognitive impairment [152]. The recommended daily dose of dry ginseng root is from 0.5 to 2 g for an extended time. Alternatively, a standardized ginseng extract (1.5–7% ginsenosides) can be taken at a dose of 200 mg per day [153].

Herbal preparations of *P. ginseng* are used worldwide, and experimental results have indicated that it might have beneficial effects on cognitive performance. In addition, it has a good safety profile, with fewer and milder side effects compared to other drugs. However, data validity is reduced by the heterogeneity of results, and variations in study duration and ginseng dosage, which makes it difficult to draw firm conclusions about *P. ginseng*’s effectiveness. Currently, there is a lack of convincing evidence to demonstrate the cognitive-enhancing effect of ginseng in healthy participants. There is also little high-quality evidence of its effectiveness in patients with dementia. Randomized, double-blind, placebo-controlled, parallel-group studies with large samples are needed to determine the effects of *P. ginseng* on cognition in different populations.

#### 3.5.6. Side Effects and Contraindications

There are few reported side effects, and those are mainly associated with overdose and include less severe problems such as diarrhea, restlessness, insomnia, headache, excessive physical stimulation, nervousness, inability to concentrate, increased or decreased blood pressure, breast tenderness, and vaginal bleeding. Ginseng appears to have neutral vascular effects, so its use should not be discouraged due to concerns about increased blood pressure. It is known that when combined with caffeine-containing substances, there is increased nervousness and possibly increased feelings of restlessness and insomnia. Ginseng can also induce asthma through an immunoglobulin-E-mediated mechanism. When ginseng is taken with alcoholic drinks, it increases the metabolism and breakdown of the ingested ethanol by the action of alcohol dehydrogenase [125,153,154,155,156,157,158].

### 3.6. Guarana (Paullinia cupana Kunth)

#### 3.6.1. History

*P. cupana* is an ancient, cultivated plant native to South America. It is better known as guarana, and the inhabitants of the South American forests have considered guarana as a plant with a healing mission since ancient times. Its name comes from the word *guaraná*, which has its origin in the Sateré-Maué word for a plant, *warana*, which in Tupi-Guarani means ‘God’s eyes.’ The plant’s fruit sometimes looks like a human eye [159,160].

#### 3.6.2. Plant Description

**Figure 6:**
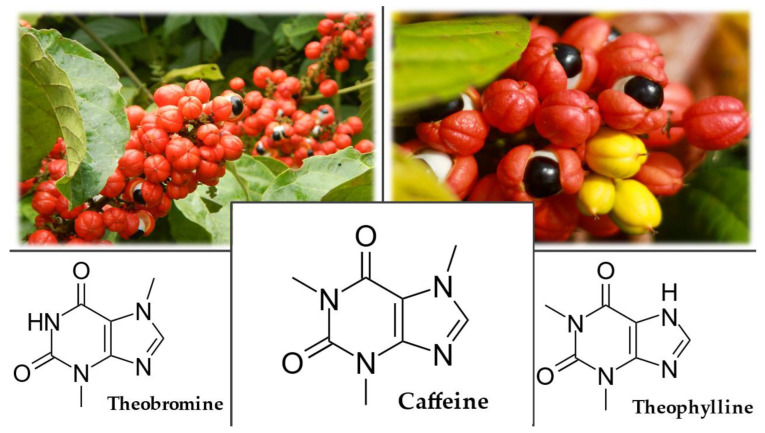
*Paullinia cupana’s* main bioactive compounds, methylxanthines, and their chemical structures.

*P. cupana* is a tropical, evergreen, climbing shrub in the Sapindaceae family. It is a vine with alternating, pinnate leaves with 3–5 smaller leaves. Its tiny flowers, white or greenish, bloom in large clusters and ripen into striking bright red fruits. The fruit is a three-capsule purse that contains one dark brown to black seed and a hazelnut-sized seed in each capsule (Figure 6). Sometimes only one seed develops [159,161].

#### 3.6.3. Occurrence

Guarana comes from the Amazon River basin, especially from its southern area. It also occurs in the Orinoco and Rio Negro River basins to a lesser extent. It usually grows wild, but recently it has also started to be produced on plantations in Brazil. The plant requires a tropical climate, heat, and humidity. In a temperate climate, the plant can only be grown in a large tropical greenhouse [159,162].

#### 3.6.4. Chemical Composition

The commercially valuable part of the plant is the seed, which has a high content of purine alkaloids of methylxanthines (caffeine, theobromine, and theophylline) (Figure 6). *P. cupana* seeds contain the world’s highest natural dose of caffeine (2 to 8%), which is 3 to 5 times higher than a coffee bean. They also contain high concentrations of tannins and other compounds such as saponins, polysaccharides (starch), proteins, fatty acids, trace elements (manganese, rubidium, nickel, and strontium), and essential oils. The plant’s essential oil contains, among other compounds, carvacrol and estragole [159,161,163].

#### 3.6.5. Uses and Nootropic or Cognitive Effects of *Paullinia cupana* Kunth

The seeds, called guarana nuts, are harvested when fully ripe, then roasted, sifted, crushed, and mixed with water to form a bitter paste with high caffeine content. After brewing this paste with hot water, a drink similar to coffee is produced. *P. cupana* paste is used to prepare non-alcoholic and alcoholic beverages, popular mainly in Brazil. The paste is dried and ground for further processing, and the resulting powder is used to make tablets [164,165].

The protective effects of a hydroalcoholic guarana extract were investigated in *Caenorhabditis elegans* models of HD and AD. The extract slowed amyloid-β-induced paralysis in a caffeine-independent manner, reduced polyglutamine protein aggregation in muscle, and polyglutamine-mediated neuronal death in sensory neurons. The protective effect of the guarana extract was associated with antioxidant activity and the modulation of proteostasis. The extract increased SOD-3 expression and proteasome activity, decreased intracellular ROS and autophagosome accumulation, and extended the animals’ lifespan [166]. The results of another in vitro study showed that guarana could prevent amyloid-β aggregation, protein glycation, and methylglyoxal-, glyoxal-, and acrolein-induced toxicity on neuron-like cells. Since these are considered pathological AD features, guarana deserves further research as a potential therapeutic agent in neurodegenerative diseases [167].

In a study on rats placed in a T-maze, a generalized anxiety and panic disorder model, the animals were given repeated doses of an aqueous fraction of *P. cupana* seeds. The results proved that guarana exhibited panicolytic and anxiolytic effects [168]. Crude lyophilized guarana seed extract had a significant nootropic effect when administered by gavage to rats over an extended time. The impact on cognitive behavior was studied using the Morris water maze test and scopolamine treatment compared to the control group. The extracts did not alter locomotor activity in the open-field test [169]. According to an in vivo experiment, mice that ingested a guarana suspension showed a significant increase in physical capacity when exposed to a stressful situation such as forced swimming. After single or repeated administration, *P. cupana* partially reversed the amnesic effect of scopolamine treatment in mice and rats as measured by the passive avoidance test, which indicated a positive impact on memory acquisition. However, in the active avoidance test, no effect was observed in rats even after 20 days of guarana administration. The treated animals had the same average lifespan as controls, indicating low toxicity of guarana after 23 months of treatment [170].

The neuroprotective effect of guarana in mouse cerebellar cells and brain neurons against vincristine exposure was investigated in an in vitro study. *P. cupana* increased cell viability, stimulated catalase activity, and reduced lipoperoxidation and ROS levels [171]. The effects of *P. cupana* consumption on erythrocyte antioxidant enzyme activity, plasma catechins, and oxidative stress biomarkers in healthy, overweight subjects were evaluated. The twelve participants received 3 g of ground guarana seeds daily for 15 days. Administration of guarana increased oxygen radical absorbance capacity in plasma, catalase, and glutathione peroxidase activity while reducing ex vivo oxidation of low-density lipoprotein and deoxyribonucleic acid damage in lymphocytes induced by hydrogen peroxide. *P. cupana* catechins were biologically available and contributed to the reduction of oxidative stress in clinically healthy individuals by direct antioxidant action and upregulation of antioxidant or detoxification enzymes [172]. In a double-blind, placebo-controlled study of 28 healthy young volunteers, the cognitive and mood effects of individual doses of 75 mg of dry ethanol extract of guarana or 200 mg of ginseng, or a combination of both, were investigated. All three treatments improved daily task performance compared to the placebo. With guarana supplementation, sentence verification and attention improvements were observed, but with reduced accuracy. The speed of performance of attention and memory tasks was increased with ginseng and the combination treatment, while all three treatments improved performance on the serial subtraction task. Due to the relatively low caffeine content (9 mg) in this dose of guarana extract, it is unlikely that these effects could be attributed to its content [173]. The guarana dosage should be 450 mg, up to five times daily for adults. Alternatively, a dose of 75 mg of guarana extract containing approximately 12% caffeine can be administered as a tablet [173,174].

Acute guarana ingestion can potentially affect specific cognitive performance, such as reaction time, performance accuracy, and secondary memory factors. However, whether such changes in performance are related to the caffeine content, other bioavailable compounds, or the potential synergy of these substances has not been confirmed. In future research, experiments and clinical trials should be carried out using guarana extracts with low caffeine content. A comparison should be made with pure caffeine, or the caffeine content of guarana should be expressed concerning body weight so that future results are comparable; also, cognitive performance tests after chronic dosing should be performed.

#### 3.6.6. Side Effects and Contraindications

Guarana can be dangerous due to its high caffeine content. Long-term use in excessive doses containing more than 600 mg of caffeine daily has been associated with side effects such as insomnia, nervousness, stomach irritation, or vomiting. Due to the relatively high content of methylxanthines (caffeine, theobromine, and theophylline), people with cardiovascular disease and those who suffer from insomnia, chronic headaches, or who use theophylline should be careful when using guarana. Recent studies have also suggested a link between the consumption of guarana polyphenols and a reduced risk of developing type 2 diabetes through the mechanism of inhibition of α-glucosidase and α-amylase activity. It is also not recommended for pregnant or breastfeeding women [175,176,177,178,179,180].

### 3.7. Maca (Lepidium meyenii Walp.)

#### 3.7.1. History

For more than 2000 years, maca has been used as a traditional food and medicinal plant. It is also known as Peruvian ginseng, although it is not botanically related to *P. ginseng* C.A. Meyer. In 1843, Gerhard Walpers named the species *Lepidium meyenii* [181,182].

#### 3.7.2. Plant Description

**Figure 7:**
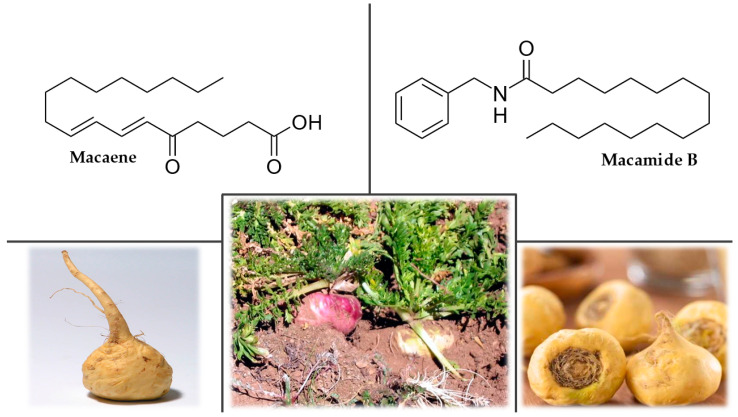
*Lepidium meyenii’s* main bioactive compounds, macamides and macaenes, and their chemical structures.

Maca is a biennial crop of the Brassicaceae family. The valuable part, the edible hypocotyl, resembles a radish in shape but is yellow and up to 8 cm in diameter (Figure 7). Several maca phenotypes characterized by different colors of their hypocotyls, from white to black, have been described. The rosette of leaves is usually sessile to the ground with a wavy edge, and the flower stalk bearing whitish flowers grows out of the center [182,183,184,185].

#### 3.7.3. Occurrence

Maca is cultivated in only one place on our planet—at altitudes of 3500–4500 m in the Andes in Peru. The locations where it is grown are mostly inhospitable with intense sunlight, strong winds, and frosts [182,183,186].

#### 3.7.4. Chemical Composition

Maca contains several bioactive chemical compounds, including glucosinolates (glucotropaeolin and glucolimnanthin), long-chain fatty acid *N*-benzylamides, macamides (*N*-benzylhexadecanamide), and unsaturated long-chain fatty acid derivatives, macaenes. Macamides and macaenes are considered characteristic markers of maca because they have not been found in any other plants (Figure 7). There are also thiohydantoins (macathiohydantoins A-K, macahydantoins A-C, and meyeniins A-C), alkaloids (imidazole alkaloids, pyrrole alkaloids, hydantoin alkaloids, and urea alkaloids), sterols (stigmasterol and sitosterols), fatty acids (caprylic, capric, lauric, myristic, palmitic, palmitoleic, stearic, oleic, linoleic, and linolenic acids) and vitamins B, C, and E. Maca is very nutritious, containing an average of 60–75% carbohydrates, 10–14% protein, 8.5% fiber, and 2.2% lipids. The dried root is rich in essential amino acids, while fresh root has unusually high iodine and iron contents. Yellow maca has a higher lipid and carbohydrate content than the red and black varieties [182,187,188,189].

#### 3.7.5. Uses and Nootropic or Cognitive Effects of *Lepidium meyenii* Walp.

The edible parts of maca are the hypocotyls and the main tap root, which, in this review, is referred to as the root to avoid confusion. The root has a distinctive flavor and aroma and is eaten either fresh or dried. In South America, the fresh roots are boiled like potatoes, and the dried roots are used to make a sweet porridge or pudding called *mazamorra de maca*. It can also be ground into flour and used like a cereal grain. A slightly alcoholic drink called *maca chica* is also produced from the plant. The leaves, stems, and flowers are also edible but remain underutilized as a food source. However, many growers mix and grind the leaves with the roots [190,191,192].

The antioxidant activity of polysaccharide fractions from *L. meyenii* leaves was investigated in vitro. It revealed a strong, dose-dependent scavenging effect on hydroxyl radicals, superoxide anions, and DPPH radicals and also showed positive results on other antioxidant parameters [193]. The biological activity of a new polysaccharide from maca leaves was investigated, and the results showed that the polysaccharide had a reducing ability and could scavenge DPPH radicals. In addition, it stimulated the immune response of a monocyte/macrophage-like murine cell line (RAW264.7). The polysaccharide also promoted proliferation, enhanced phagocytosis, and increased NO production in a dose-dependent manner [194]. 

The neuroprotective activity of the maca plant was studied both in vivo and in vitro. In the latter case, cultured crayfish neurons were pre-treated with a vehicle or a pentane extract of *L. meyenii* and exposed to hydrogen peroxide. Cell viability was determined microscopically and chemically, and a significant concentration-dependent neuroprotective effect was demonstrated. The pentane extract was next administered intravenously to rats before and after middle cerebral artery occlusion. While infarct volumes were reduced at the lower dose, higher doses increased infarct volumes compared to controls [195]. *L. meyenii* compounds showed potent neuroprotective activity in the 1-methyl-4-phenyl-1,2,3,6-tetrahydropyridine (MPTP)-induced zebrafish model of Parkinson’s disease. The significant neuroprotective action of the methanol fraction was probably mediated by the inhibition of acetylcholinesterase and butyrylcholinesterase [196]. These results suggest a potential application of *L. meyenii* Walp. as a neuroprotectant.

In another in vivo experiment, maca showed antidepressant activity and beneficial effects on latent learning in ovariectomized mice. It also reduced immobility time in the swimming strength test [197]. *L. meyenii* further improved motor coordination, endurance, and cognitive function in 14-month-old male mice that received maca powder by gavage for five weeks. This effect may be associated with the upregulation of autophagy-related proteins in the cortex and enhanced mitochondrial respiratory function [198]. In another in vivo study, a neurotoxic model was established by subcutaneous corticosterone injection for 21 days, and the effects of macamide against corticosterone-induced neurotoxicity were tested. Experiments showed that macamide exhibited anti-inflammatory, neurotrophic, and synapse-protective properties, reduced hippocampal neuroinflammation, alleviated depressive-like behavior, and improved hippocampal neurogenesis and neurotrophy [199]. 

A randomized, placebo-controlled, double-blind study was conducted on 60 adult women who consumed maca extract or placebo for four weeks. The effect of maca extract containing benzyl glucosinolate (9.6 mg/day) on fatigue was evaluated, and there was a significant reduction in fatigue compared to before consumption. Thus, *L. meyenii* supplements containing benzyl glucosinolate represent a potential anti-fatigue treatment [200]. These findings suggest that *L. meyenii* could provide a beneficial functional food in the future to slow age-related cognitive decline. The optimal dose has not been determined, but 1.5 to 3 g of dried maca root per day for an adult was effective in these studies with no observed side effects [201,202].

In vitro and in vivo experiments have revealed various nootropic effects of *L. meyenii* products, such as memory and learning enhancement, reduced fatigue, and neuroprotective activity. The studies that have been conducted have shown that maca has enormous potential, but further research is needed to prove its safety and efficacy. The relationship between the compounds and their biological activity needs to be better described, tests at the molecular level should be performed, and their lack of toxicity as phytotherapeutic agents should be verified. More clinical trials are needed to provide a clear answer regarding the beneficial effects of *L. meyenii* on cognitive functions.

#### 3.7.6. Side Effects and Contraindications

Maca has shown low levels of cellular toxicity in vitro and acute oral toxicity in animals. Liver damage was observed in a 30-year-old man ten days after drinking 300 mL of medicinal *L. meyenii* liquor containing 50% (*v*/*v*) alcohol. In contrast, methanolic and aqueous maca extracts up to 10 mg/mL showed no cytotoxicity in primary hepatocyte cultures. The LD_50_ for rats was 17 g/kg body weight, and no toxicity was measured with a single acute dose of maca extract. In the future, it will be necessary to develop a scientific approach to evaluating maca’s promising biological activity in humans and to determine a safe dosage range based on body size [203,204,205,206].

### 3.8. Rhodiola (Rhodiola rosea L.)

#### 3.8.1. History

A 1200-year-old Tibetan dictionary describes the healing properties of this mountain plant. Beginning at the end of the 1980s, the demand for rhodiola as a raw material for drug preparation has steadily increased. The quality of wild plant material has fluctuated considerably in the past, so its cultivation has begun. According to historical sources and traditional recipes in Europe, it was first used in folk medicine in Russia and Scandinavia. The Vikings used the plant to bolster their physical strength and endurance under adverse conditions. This plant was mentioned in a Swedish herbal pharmacopeia and was even known and described by the Greek physician Dioscorides in his *De Materia Medica* [207,208].

#### 3.8.2. Plant Description

**Figure 8:**
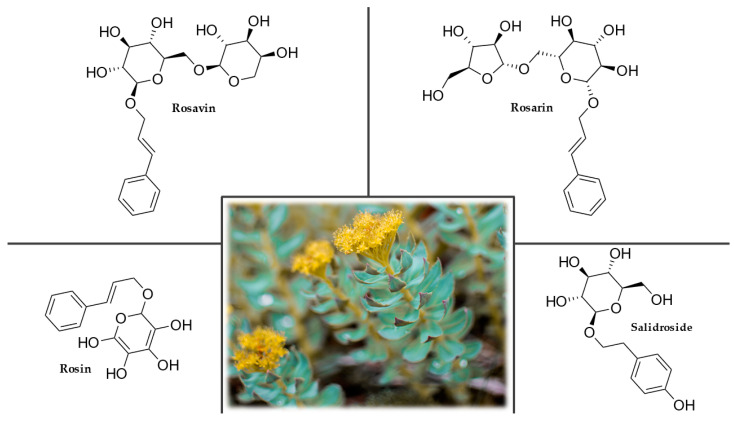
*Rhodiola rosea’s* main bioactive compounds, rosavins and salidroside, and their chemical structures.

Rhodiola, commonly called golden root, is a woody, frost-resistant perennial in the Crassulaceae family. The plant’s height is usually 25–30 cm, but it can reach 60 cm under ideal conditions. The aboveground herbaceous shoots die back every year. Below ground, it has a thick, branched root from which green or reddish buds emerge. The root is gray-yellow, pinkish inside when cut, and after being ground, it smells of roses, hence the species name of the plant, *rosea*. In the spring, the buds grow shoots with alternately sessile, flat, triangular leaves, 0.7–3.5 cm long, 0.5–1.5 cm wide, elliptically elongated, and sharp at the tip with a few teeth. The inflorescence appears at the end of spring or the beginning of summer and is composed of a cluster of four florets. The corolla is yellow in male flowers, brownish-red in females, and stunted (Figure 8). The fruits are reddish or greenish with many seeds, which are tiny, brown, egg-shaped, and ribbed [209,210].

#### 3.8.3. Occurrence

Rhodiola is a manganophilic (manganese-concentrating) plant that occurs up to 2500 m above sea level in nature. It grows mainly in the Northern hemisphere, in the mountains and highlands of the Far East, the Carpathians, western, central, and southern Europe, Asia Minor, Mongolia, northern China, Canada, and Greenland. The plants occur mainly in moist rock crevices, rubble, rocky slopes, embankments, and wet soils along river banks. This plant’s abundance has decreased significantly due to intensive collection. *R. rosea* is today listed as a protected plant and has been registered by the International Union for Conservation of Nature (IUCN) on its Red List of threatened species [207,208,211].

#### 3.8.4. Chemical Composition

Six groups of active substances can be found in the roots and rhizomes of rhodiola. The important ones are cinnamyl alcohol glycosides, rosavins (rosavin, rosarin, and rosin), tyrosol glucosides, and salidroside (or rhodioloside) (Figure 8). Flavonoids (rhodionin and rhodiosin), monoterpenes (rosiridol and rosidarin), and triterpenes (daucosterol and β-sitosterol) are also important. Rhodiola contains many organic acids, particularly citric, tartaric, malic, succinic, fumaric, chlorogenic, and gallic acids. These substances all contribute to the specific activity of the extracts. The increased content of manganese (up to 0.8%) and tannins (up to 20%) is also unusual [207,209,212].

#### 3.8.5. Uses and Nootropic or Cognitive Effects of *Rhodiola rosea* L.

The above-ground plant parts are traditionally consumed as a source of edible vegetables in the spring. The green leaves are eaten as a salad or dipped in molasses and sometimes vinegar. Early spring buds are said to resemble Brussels sprouts, and the stems are often cooked with pork. The roots are sweet and can be eaten raw or cooked as a meal. Roots and rhizomes from older plants are also collected, dried, and used to produce the extract [213,214].

The results of an in vitro study on neuronal cell lines overproducing amyloid-β showed that salidroside, a phenylpropanoid glycoside isolated from *R. rosea*, reduced the induced cytotoxicity and attenuated the intracellular accumulation of ROS and malondialdehyde by activating antioxidant enzymes in a dose-dependent manner [215]. Similar conclusions were reached in another in vitro study in which salidroside demonstrated a broad spectrum of pharmacological properties in a cultured rat pheochromocytoma cell line (PC12). Experimental results indicated that it protected cells against hypoglycemia and cytotoxicity induced by serum restriction by modulating apoptosis-related gene expression, restoring mitochondrial membrane potential, and inhibiting intracellular ROS production [216].

Another experiment aimed to determine whether salidroside could improve the survival of mesenchymal stem cells. This technique is considered a promising treatment for alleviating cerebral ischemic damage. Cells were pre-treated with salidroside under hypoxic–ischemic conditions and subsequently transplanted into rats after middle cerebral artery occlusion. The results showed that salidroside promoted proliferation and migration and reduced cell apoptosis under hypoxic–ischemic conditions. In vivo experiments revealed that transplantation of cells pretreated with salidroside enhanced the therapeutic efficacy by increasing neurogenesis and inhibiting neuroinflammation in the hippocampal CA1 region after ischemia [217]. A similar experiment was performed on long-term cultured rat hippocampal neurons and aged mice treated with salidroside. The results showed that salidroside increased the viability and expression of microtubule-associated protein 2 and decreased β-galactosidase levels in rat neurons. Salidroside reduced cognitive dysfunction, attenuated neuronal degeneration in the CA1 region, and lowered oxidative stress levels in aging mice. Salidroside also promoted the expression of telomerase reverse transcriptase protein via the phosphatidylinositol 3-kinase/protein kinase B (PI3K/Akt) pathway. Salidroside treatment could be an effective strategy to increase cell survival and prevent cerebral ischemic injury and aging-related diseases [218].

An in vivo experiment was conducted in mice to investigate the effects of a single oral dose of a hydroalcoholic extract of *R. rosea* containing 3% rosavin and 1% salidroside on the CNS. The extract was tested for anxiolytic, antidepressant, adaptogenic, nociceptive, and locomotor activity at various doses using predictive behavioral tests. The results showed that the extract induced anxiolytic, antidepressant, adaptogenic, and stimulating effects that were not dose-dependent [219]. A double-blind, randomized, placebo-controlled trial with a repeated low-dose regimen investigated the normalizing and stimulating effect of *R. rosea* extract in international students during an examination period. The students used the extract or placebo for 20 days. Improvements in the *R. rosea* extract group were observed in physical fitness, neuromotor tests, mental fatigue, and overall well being; however, no significant improvement was observed in the neuromuscular tapping test and the correction of text test [220]. A randomized trial evaluated the effect of *R. rosea* extract on stress, anxiety, cognition, and other symptoms. Eighty mildly anxious human participants were randomized into two groups receiving either a dose of the extract or no treatment. Compared to the control group, the experimental group showed significant reductions in stress, anger, anxiety, confusion, depression, and overall mood improvement after two weeks. However, no relevant differences in cognitive performance were observed between the groups. Although this was a non-placebo-controlled study, it is unlikely that the findings resulted from placebo effects because the changes occurred gradually and were specific to certain psychological measures [221]. The daily dose for an adult of *R. rosea* extract for long-term use should be from 360 to 600 mg, standardized to a content of 1% rosavin [222].

Rhodiola has established uses as an adaptogen in traditional medicine in Nordic, Eastern European, and Asian countries for increasing mental and physical performance, as an antioxidant, and for reducing fatigue and depression. From the evidence presented so far, it can be concluded that there is encouraging support for the beneficial effect of *R. rosea* on cognitive function and fatigue, as demonstrated by numerous preclinical in vitro and in vivo experiments and several clinical trials. It has also been shown that rhodiola exerts preventive and protective effects by suppressing oxidative stress in neuronal tissue, which could be potentially helpful in treating AD patients. However, there have been few clinical trials in humans, so it may be difficult to determine the therapeutic dosage range and confirm the absence of adverse effects. It is also recommended that future research focus on identifying the neuroprotective components of *R. rosea* using new phytochemical analysis techniques to clarify the molecular mechanisms of its neuroprotective effects.

#### 3.8.6. Side effects and Contraindications

The most common side effects include gastrointestinal problems, headache, restlessness, nausea, and insomnia. So far, there has been insufficient clinical data to determine the safety of its use during pregnancy and breastfeeding [223,224].

### 3.9. Schisandra (Schisandra chinensis Turcz. Baill.)

#### 3.9.1. History

*S. chinensis*, sometimes called magnolia vine or Chinese magnolia vine, has been used in China as a medicine and ‘harmonizer’ since ancient times [225]. Schisandra was first mentioned in *Shennong Ben Cao Jing*, written over 4000 years ago. Around 1850, schisandra was brought to European botanical gardens in eastern Russia. Its original botanical name *Kadsura chinensis*, was later changed to *Maximowiczia chinensis*, and then established as *Schisandra chinensis*. The genus name, *Schisandra,* combines the Greek words *schizein*, which means ‘to split’, and *andros*, which means ‘man’, which refers to the fact that the plant has separate male and female flowers (monoecious) [85,226].

#### 3.9.2. Plant Description

**Figure 9:**
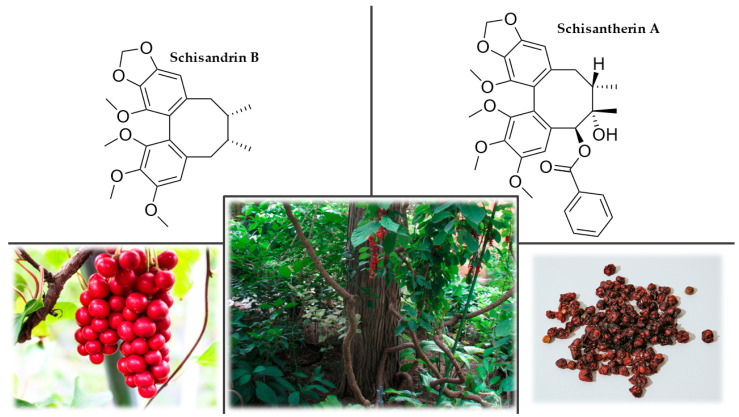
*Schisandra chinensis’s* main bioactive compounds, schisandra lignans, and their chemical structures.

Schisandra is a deciduous, climbing woody creeper with a clockwise winding stem. The stalk is 2–8 m long and usually winds around a support. The buds are 3–5 mm long, longitudinally ovate, pointed in shape, deviated from the shoot, and form in threes in the inflorescence. The leaves are alternate, dark green, and elliptical, with long, reddish petioles. The plant is monoecious, and its flowers are unisex. They are 1–1.5 cm in diameter, on long flower axes, waxy, white, yellow, or even red. It has a sweet scent. Male flowers have white stamens and are located near the bottom of the shrub. The pistillate flowers are located in the upper part of the shrub on stems with greenish scars and numerous leaves. After pollination, the ovary lengthens, takes on the shape of a grape, and produces 5–40 berries. The fruits are clustered in a group of numerous single-seeded and double-seeded red berries (Figure 9). The berries are 5–7 mm in diameter, soft, and slightly elongated. Each berry has 1–2 kidney-shaped seeds up to 3 mm in diameter. Ripe fruit is usually very juicy and has a citrus taste, hence its Russian name *limonnik*. The skin is sweet, the flesh is sour, and the seed is bitter with an essential oil flavor. This is also reminiscent of the Chinese name *wuweizi*, or ‘fruit of the five flavors’. According to various authors, the *Schisandra* genus includes 20 to 30 species [226,227,228,229,230].

#### 3.9.3. Occurrence

Schisandra comes from Asia and occurs naturally around the Amur River in southern Sakhalin, the Kuril Islands, Japan, and northern China. It grows mainly in mixed forests, mainly on alluvial, slightly permeable soils along rivers at an altitude of 200–500 m. It tolerates moist soils but does not grow with permanent wetting or flooding or on sandy or muddy soils. Schisandra is a light-loving plant, but it can grow in the shade while young. Later, however, a shaded plant will bear little fruit. Its preferred growth, and cultural conditions are characterized by high resistance to winter and cold weather. It is often planted in western European gardens as an ornamental [226,227].

#### 3.9.4. Chemical Composition

The active components include dibenzocyclooctadiene lignans (schisandrins B and C, schisantherins A and B, schisanchinins A-D, schisanthenol, deoxyschisandrin, and gomisins A and G). Because their occurrence is limited to this species, these compounds are often called schisandra lignans (Figure 9). The amount of these compounds in the fruit is affected by its degree of ripeness, the time of harvest, the habitat, and location. In addition, other types of lignans, dibenzylbutane lignans (schineolignans A-C) and tetrahydrofuran lignans (schinlignins A and B), were isolated from the fruits. Among the triterpenoids, pre-schisanartanins A-C and wuweizilactone acid are represented, and in the monoterpenes, borneol, eucalyptol, α-pinene, and β-pinene were identified. In the sesquiterpene group, schisandra contains sesquicarene. Schisandra also contains flavonoids (hyperoside, isoquercitrin, rutin, and quercetin), and fumaric, citric, malic, ascorbic, tartaric, succinic, chlorogenic, p-coumaric, p-hydroxybenzoic, salicylic, and syringic acids, vitamins C and E, and the phytosterols, stigmasterol, and β-sitosterol. The seeds contain 33% oil, composed of linolenic acid and oleic acid glycerides. The whole plant is permeated with essential oil, about 3% in the bark and 2% in the seeds [228,231,232].

#### 3.9.5. Uses and Nootropic or Cognitive Effects of *Schisandra chinensis* (Turcz.) Baill.

The fruits are most often dried and preserved in honey or sugar. They can also be processed to make tea, syrup, jam, compote, and a strong sweet wine from the fermented juice. A tincture can also be prepared from crushed seeds, dried berries, or leaves [233,234,235].

The antioxidant and immunological activities of polysaccharide extracts of *Schisandra sphenanthera* and *S. chinensis* were investigated in vitro. *S. sphenanthera* extracts showed more remarkable free radical scavenging ability, protective effects on biomolecules, and cellular antioxidant activity, probably due to higher protein and uronic acid contents. Conversely, regarding cell viability, phagocytosis, NO production, and acid phosphatase activity, a *S. chinensis* extract showed more potent effects because of its high levels of glucose, mannose, galactose, and arabinose [236]. An in vivo study investigated the neuroprotective effects of *S. chinensis* compounds against D-galactose-induced neurotoxicity in rats. Rats were injected subcutaneously with D-galactose and orally administered an aqueous extract or a 95% ethanol extract of *S. chinensis* fruits for six weeks. Changes in cognitive function were assessed using the step-down passive avoidance test and the Morris water maze. The results showed that the extract improved the induced cognitive deficit and partially reversed the effects of reducing the activity of SOD, catalase, and total antioxidant effect induced by D-galactose and maintained normal levels of glutathione, malondialdehyde, and NO in serum, prefrontal cortex, striatum, and hippocampus [237]. The preventive effects of schisandrin B on scopolamine-induced dementia in mice were also studied. Scopolamine increased nitrite levels and oxidative stress in the brain and decreased antioxidant enzyme levels. Subsequently, the passive avoidance test and Morris water maze showed significant memory and learning impairment, with a simultaneous increase in acetylcholinesterase activity and a decrease in acetylcholine levels. Schisandrin B pretreatment prevented scopolamine-induced oxidative stress, improved behavioral tasks, suppressed the increase in acetylcholinesterase activity, and maintained acetylcholine levels [238].

The results of in vitro and in vivo tests showed that the dibenzocyclooctadiene lignan, schisantherin A, exhibited neuroprotective effects on human neuroblastoma cells (SH-SY5Y) incubated with 1-methyl-4-phenylpyridinium ions, and in vivo on MPTP-induced PD mice. Pretreatment with schizantherin A inhibited the induced cytotoxicity in SH-SY5Y cells and reduced MPTP-induced damage in dopaminergic neurons in the PD mouse model. This neuroprotective activity was partially mediated through the regulation of two distinct pathways, including an increase in B-cell lymphoma 2 protein expression mediated by cyclic adenosine monophosphate-response element-binding protein and the activation of PI3K/Akt survival signaling [239]. A similar in vitro and in vivo study also demonstrated the neuroprotective effects of schisantherin A in multiple experimental models of Parkinson’s disease. Schisantherin A pretreatment protected against selective nerve damage by reducing intracellular ROS accumulation and inhibited NO overproduction by reducing overexpression of inducible NO synthase in SH-SY5Y cells treated with the dopaminergic neurotoxin 6-hydroxydopamine. In vivo, schisantherin A restored locomotor behavioral deficits and prevented dopaminergic neuron loss in zebrafish. Thus, it is a promising agent for preventing and treating neurodegenerative diseases associated with oxidative stress [240].

Schisandra first gained recognition as an adaptogen in the official medicine of the Soviet Union in the early 1960s, mainly due to many pharmacological and clinical studies. Russian scientists evaluated its pharmacological effects on humans, including neurosis and physical and mental work capacity. However, there has been no clinical trial for a specific neurodegenerative disease [233]. The daily dose of dried *S. chinensis* fruits for adults is between 2 and 6 g, optimally up to 0.1 g of fruit per kg of body weight [233,241].

In traditional medicine, schisandra is used to treat various disorders and diseases. *S. chinensis* lignans showed potential cognitive and neuroprotective enhancement and improvement mainly through antioxidant, anti-apoptotic, and anti-inflammatory mechanisms, modulation of various signaling pathways, and improved energy metabolism in the brain. Based on the accumulated data, it can be concluded that schisandra lignans can serve as promising neuroprotective agents by regulating various pathophysiological processes. An advantage of using *S. chinensis* extract is the apparent lack of acute or cumulative side effects. Although schisandra extracts and individual lignan compounds showed strong neuroprotective properties in vitro experiments and in animal models, almost no clinical trials against neurodegenerative diseases have been conducted. Therefore, future studies on humans are essential for developing safe and effective neuroprotective drugs from *S. chinensis*.

#### 3.9.6. Side Effects and Contraindications

No serious side effects were reported. Minor side effects included heartburn, stomach upset, and decreased appetite. Restlessness and insomnia may occur after consuming excessive amounts of the fruit. Due to a lack of safety data, schisandra should not be used during pregnancy, breastfeeding, or by children [242].

### 3.10. Water Hyssop, Brahmi (Bacopa monnieri (L.) Wettst.)

#### 3.10.1. History

In Southern India, *B. monnieri* has been used in Ayurvedic medicine for 3000 years under the name brahmi. The name is derived from the Sanskrit word *brahma*, which means ‘god’ in the Hindu religion, the ubiquitous creative power considered the equivalent of Prajapati [243,244,245].

#### 3.10.2. Plant Description

**Figure 10:**
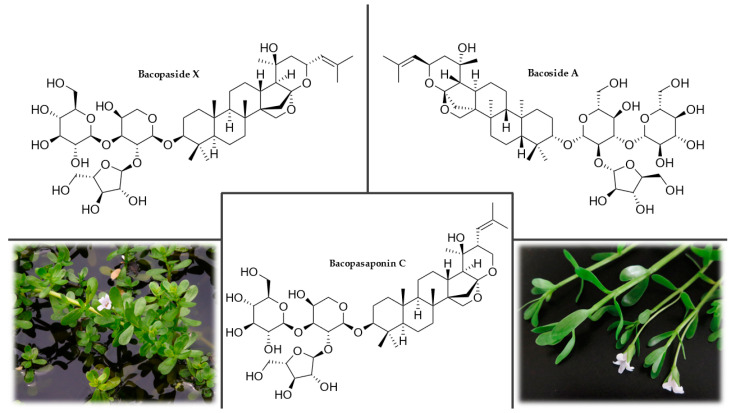
*Bacopa monnieri’s* main bioactive compounds, bacopasides, bacopasaponins, and bacosides, and their chemical structures.

Bacopa is a creeping, perennial herb of the family Plantaginaceae (formerly Scrophulariaceae), which usually grows to a height of 10–30 cm. The plant has branched recumbent or ascending fleshy stems with small sessile leaves (Figure 10). The leaves are axillary, ovate to ovate-wedge-shaped, fleshy, 0.5–2.5 cm long, and 3–8 mm wide. The flowers grow in the troughs of the leaves. They are five-petaled, purple-blue, or white with darker veins. It is often confused with *Centella asiatica* (L.) Urban, as both these plants are known by the same Hindi name, brahmi, in many places in India [246,247,248,249].

#### 3.10.3. Occurrence

The plant is widespread in the tropics and subtropics of Africa, Asia, Australia, and America. It is known in Nepal, Sri Lanka, China, Taiwan, and Vietnam. It grows in humid, muddy, flooded places and often in running water. Sometimes it is also grown as an aquarium plant. In a temperate climate, *B. monnieri* is usually an annual or houseplant [246,247].

#### 3.10.4. Chemical Composition

Water hyssop contains several compounds, the most important of which are triterpene dammarane type saponins, called bacosides A, B, and C, bacopasaponins A, B, C, and D, and bacopasides (Figure 10). Among the alkaloids, brahmine, nicotine, and herpestine are represented. Among the steroids, such as stigmasterol and β-sitosterol, the plant also contains flavonoids such as apigenin and luteolin. Other substances include hersaponin, monnierasides, cucurbitacins, and plantainoside B [250,251].

#### 3.10.5. Uses and Nootropic or Cognitive Effects of *Bacopa monnieri* (L.) Wettst.

Fresh *B. monnieri* (brahmi) leaves are commonly used as a vegetable in salads or soups, but when dried and ground, they can be freely added to any food or drink to increase their nutritional value [252,253].

In vitro treatment of rat astrocytes with a brahmi methanol extract significantly reduced the damage caused by high NO concentrations. Glial cells produce NO when stimulated by superoxide radicals [254]. A hexane extract of brahmi prevented glutamate toxicity in HT-22 cells by reducing ROS production and preventing mitochondrial and endoplasmic reticulum dysfunction through oxidative stress [255]. The nootropic effects of *B. monnieri* extract have also been demonstrated in vitro in mouse brain homogenates. The extract was found to reduce divalent metal ions, dose-dependently scavenge ROS, inhibit lipoxygenase activity, and reduce lipid peroxidation [256,257]. Thus, the results suggested that the extract possessed antioxidant activity. 

Studies in transgenic mice with mutations in human presenilin 1 and amyloid-β precursor protein that spontaneously cause the formation of amyloid plaques have shown that both short-term and long-term treatments with *B. monnieri* reduced the amount of amyloid-β found in different brain regions [258]. The effect of an alcohol extract of brahmi on the expression of choline acetyltransferase in the hippocampus was studied in mice with olfactory bulbectomy, which reduces cholinergic activity. The results showed that extract administration gradually improved cognitive dysfunction [259]. In a rat model of AD, brahmi alcohol extract also improved escape latency in the Morris water maze test and attenuated neuron loss [260]. Treatment of rats with an alcohol extract of brahmi caused an increase in hippocampal protein and protein kinase activity that resulted in enhanced cognitive functions such as memory and learning. The chemical compounds identified as responsible for this positive effect were a mixture of two saponins, bacosides A and B [261]. The reported pro-cognitive effect of *B. monnieri* on rats may be partially explained by an observed increase in cerebral blood flow independent of a change in blood pressure [262]. 

Experiments have shown a significant effect of *B. monnieri* on the ability to process new information. To test this, 76 volunteers between the ages of 40 and 65 participated in a double-blind, randomized trial of a brahmi extract in which memory function was assessed, and anxiety levels were measured. The learning ability was unchanged, but the forgetting process was significantly reduced [263]. A group of researchers reported that doses of brahmi and *Sideritis scardica* (mountain tea) extracts given to ten elderly human subjects with mild cognitive impairment led to improvements in a d2-concentration test. Differences were also documented by quantitative electroencephalography mapping [264]. A randomized, double-blind, placebo-controlled trial was conducted on 28 healthy adults over the age of 55 who were required to complete cognitive training for three hours per week for three months. Fifteen of the subjects consumed a standardized *B. monnieri* extract daily, and thirteen consumed a placebo. Neuroimaging results indicated an improvement in neural network complexity in those receiving the combination of Brahmi and cognitive training, but most results did not reach statistical significance [265]. The daily recommended dosage of brahmi extract standardized to 20% bacosides A and B is 100–200 mg in divided doses for children and 200–400 mg in divided doses for adults [245,266].

Although animal experiments have shown that *B. monnieri* has nootropic effects in established models of learning and memory, the exact mechanism of its action is still uncertain because its secondary metabolites constitute a kind of active pharmacological complex. Brahmi, administered over an extended period, improved information processing, verbal learning, and memory consolidation in humans, but no acute effects on cognitive function were found at lower doses. Most clinical trials have also primarily examined the effects of brahmi on memory. Other cognitive activities such as auditory perception, numeracy, and language learning have been studied very little or not at all. Therefore, further clinical studies must be carried out to investigate the possible side effects and contraindications.

#### 3.10.6. Side Effects and Contraindications

*B. monnieri* has a high therapeutic index, which is responsible for its safety during long-term use. No serious side effects have been reported so far. Rarely, nausea, sedation, or gastrointestinal distress may occur after ingestion. The LD_50_ for rats is 2500 mg/kg. There are no studies regarding the safety of this herbal preparation in pregnant women, so it should not be used during pregnancy [267,268,269].

## 4. Conclusions and Future Perspectives

Nootropic plants used in traditional medicine are a heterogeneous group, including representatives of all types of plants. In contrast to synthetic nootropics, natural plant extracts have variable phytochemical compositions that can synergistically affect the metabolism of neurons in the CNS and improve cognitive function, especially in cases of neuronal damage or degeneration. They are used in chronic, subacute, and acute disorders of consciousness, memory, and learning and as supportive treatments in patients with senile dementia, Alzheimer’s, and Parkinson’s disease. Most nootropic plant drugs are not immediately effective after a single administration and must be taken at optimal doses for an extended time before measurable improvement occurs. Another problem in research on natural substances and their products is the standardization of form and dosage. It is difficult to compare results from different laboratories without addressing this problem, which is particularly important when studying the effects of plant compounds. Plants contain many substances that can cause diverse biological effects, depending not only on the genotype but also on the place of their growth because the soil composition, climate, and the overall supply of nutrients can affect the chemical composition of the plant tissues. In addition, it must be kept in mind that the nootropic, cognitive, neuroprotective, and antioxidant properties are not produced by a single molecular species but by a synergistic combination of different compounds. The effects produced by a single isolated compound may have little in common with the effects produced by a full spectrum extract from that plant. Plant nootropics are generally very well tolerated, but potential users should consider their overall health condition and consult a doctor about possible contraindications and drug interactions before trying a particular plant formulation. However, if the recommended dosage is followed, no serious complications should occur because side effects are rare and usually mild. There are not enough experimental and clinical studies on using these plant extracts during pregnancy and breastfeeding, and caution is advised. For the same reason, administration to children is not recommended. Future research on plant-based nootropics should focus on double-blind, randomized, multicenter clinical trials with more diverse groups regarding gender, age, and health status. In addition, advanced methods based on neuroimaging assessment should be included in trials, studies, and experiments to evaluate any potential beneficial effects. Future searches for nootropic plants will undoubtedly reveal additional candidates and combinations.

## Data Availability

Not applicable.
